# Evaluation of Changes in *Staphylococcus* and *Enterococcus* Resistance Following Reduced Restrictions on Daptomycin and Linezolid

**DOI:** 10.1093/ofid/ofag523

**Published:** 2026-08-22

**Authors:** Blake A Jennewein, Karrine D Brade, Meghan N Jeffres, Garth C Wright, Douglas N Fish

**Affiliations:** Department of Pharmacy, UCHealth University of Colorado Hospital, Aurora, Colorado, USA; Department of Clinical Pharmacy, University of Colorado Skaggs School of Pharmacy and Pharmaceutical Sciences, Aurora, Colorado, USA; Department of Pharmacy, UCHealth University of Colorado Hospital, Aurora, Colorado, USA; Department of Pharmacy, UCHealth University of Colorado Hospital, Aurora, Colorado, USA; Department of Clinical Pharmacy, University of Colorado Skaggs School of Pharmacy and Pharmaceutical Sciences, Aurora, Colorado, USA; Department of Clinical Pharmacy, University of Colorado Skaggs School of Pharmacy and Pharmaceutical Sciences, Aurora, Colorado, USA; Department of Clinical Pharmacy, University of Colorado Skaggs School of Pharmacy and Pharmaceutical Sciences, Aurora, Colorado, USA

**Keywords:** antimicrobial resistance, coagulase-negative staphylococci, daptomycin, enterococcus, linezolid, MRSA, *Staphylococcus aureus*, VRE

## Abstract

**Background:**

Daptomycin and linezolid may have more favorable safety and efficacy profiles compared to vancomycin and require less therapeutic monitoring. To capitalize on these advantages, the University of Colorado Hospital relaxed prescribing restrictions and updated local guidance for the treatment of Gram-positive infections, resulting in increased daptomycin and linezolid utilization. The objective of this study was to assess the impact of liberalized daptomycin and linezolid use on minimum inhibitory concentrations (MICs) in Gram-positive organisms.

**Methods:**

This retrospective, single-center study included all *Staphylococcus* and *Enterococcus* isolates across an 8-year period, including a 3-year follow-up period after implementing practice changes. Organisms were categorized as hospital-acquired (HA) if the culture was collected 48 hours after admission. Interrupted time series analyses were performed to measure changes in geometric mean (GM) of MICs before and after implementation.

**Results:**

Minimum inhibitory concentrations from 19 160 isolates were evaluated, of which 7224 were HA. Linezolid use increased to 43.7 days of therapy per 1000 patient days (DOT/1000PD), and daptomycin use increased to 15.7 DOT/1000PD post-implementation. Linezolid GM MICs trended downward pre-implementation, and the trajectory was unaffected by implementation except in coagulase-negative *Staphylococcus* (CoNS). Daptomycin GM MICs declined pre-implementation among *Enterococcus*, but not *Staphylococcus.* Implementation was associated with a significant slope change in vancomycin-resistant *Enterococcus* (VRE) and HA CoNS. Resistance rates and MIC distributions were not impacted by implementation.

**Conclusions:**

The liberalization of daptomycin and linezolid did not result in clinically relevant increases in resistance. Further studies are needed to confirm the durability of these results, specifically among CoNS and VRE.

## Teaser

The preferential utilization of daptomycin and linezolid over vancomycin for Gram-positive infections at a large academic medical center was not associated with clinically significant increases in staphylococcal or enterococcal resistance rates across an 8-year study period.

The development of antimicrobial resistance (AMR) is one of the most critical global public health threats today [1, 2]. In 2019 alone, AMR was responsible for 1.27 million deaths worldwide, including over 35 000 in the United States [3, 4]. Gram-positive resistance is particularly concerning due to the increased virulence and mortality associated with organisms such as *Staphylococcus aureus* [5–7]. *S. aureus* is the leading bacterial cause of death in 135 countries and is individually responsible for over 1 million deaths globally each year [8]. Despite recent strides in infection control measures, methicillin resistance rates in *S. aureus* remain high and continue to increase each year at many institutions [4, 9, 10]. Infections caused by *Enterococcus* species are also concerning because of their intrinsic resistance and opportunistic pathogenicity [11, 12]. They are frequently implicated in hospital-acquired infections leading to mortality rates as high as 33.5% in some reports [8, 11, 13, 14]. Consequently, the CDC has designated methicillin-resistant *S. aureus* (MRSA) and vancomycin-resistant *Enterococci* (VRE) as serious threats to public health because of their propensity to develop further resistance [4].

Given the increasing burden and severity of these infections, judicious stewardship of limited antibiotic options is crucial to preserve their activity and delay further development of AMR. Vancomycin has long been considered the standard of care for MRSA and *Enterococcus* infections [15, 16]. Newer agents such as daptomycin and linezolid are also used for these pathogens and retain activity against vancomycin-resistant organisms [17]. Conventionally, vancomycin is recommended for these infections to lessen the selective pressure for daptomycin and linezolid resistance [16, 18–20]. However, a growing body of literature reports lower rates of mortality and clinical failure for MRSA bloodstream infections with daptomycin compared to vancomycin [21–25]. Linezolid has also demonstrated superiority over vancomycin, particularly in respiratory tract and skin and soft tissue infections [26–30]. In addition, vancomycin carries significant challenges in terms of pharmacokinetic dosing, system implementation, and toxicity [31–35].

Considering these data, some institutions are shifting to preferentially utilize daptomycin and linezolid in many clinical scenarios; however, the epidemiological impact of this practice on resistance is largely unknown. Although resistance to these agents appears low overall among both *Staphylococcus* and *Enterococcus* [36, 37], small studies have shown an association between daptomycin or linezolid use and subsequent resistance [38–43]. The aim of this study was to characterize the impact of increased utilization of daptomycin and linezolid on staphylococcal and enterococcal minimum inhibitory concentrations (MICs) over an 8-year period, including a 3-year follow-up period after implementation of institutional policy changes regarding antibiotic prescribing at a large academic medical center.

## METHODS

### Study Setting and Design

A retrospective analysis of clinical isolates and antibiotic utilization was conducted at the University of Colorado Hospital (UCH) from 1 January 2016, to 30 April 2024. University of Colorado Hospital is an 800-bed academic medical center and a quaternary referral site for the region, including 15 other hospitals within the University of Colorado Health system. The patient population at UCH includes immunocompromised individuals such as solid organ and stem cell transplant recipients. This study was approved by the Colorado Multiple Institutional Review Board.

### Changes to Local Prescribing Practice

University of Colorado Hospital employs a criteria-based restriction system to protect select antibiotics. Prior to 2019, UCH routinely favored vancomycin for empiric and definitive coverage of resistant Gram-positive organisms such as MRSA and *Enterococcus*. Additionally, daptomycin and linezolid use was restricted exclusively to approval by infectious disease or antimicrobial stewardship services. In July 2019, local guidance for the management of skin and soft tissue infections was updated to recommend linezolid as the preferred anti-MRSA agent for empiric and definitive treatment. Additionally, a hard stop for the concomitant use of linezolid and serotonergic medications was changed to a warning during the medication ordering and verification process in the electronic medical record. In December 2019, UCH order sets were further updated to include linezolid as the preferred empiric and definitive anti-MRSA agent for respiratory tract infections requiring MRSA coverage. A protocol allowing pharmacists to independently order MRSA nasal swabs was implemented the following year. In 2021, treatment guidance for *Staphylococcus* spp. bloodstream infections was updated to recommend daptomycin as first-line therapy for definitive treatment. Coupled with each of these guidance updates, corresponding antibiotic restriction criteria were also relaxed for each agent. We refer to this joint intervention of removing prescribing protections and updating local guidance documents and order sets as liberalization. Following liberalization, antimicrobial stewardship services continued to monitor both daptomycin and linezolid appropriateness with the revised preapproval criteria and ongoing prospective monitoring and feedback.

### Microbiological Data

Monthly antimicrobial use data for vancomycin, daptomycin, and linezolid were collected as days of therapy per 1000 patient days (DOT/1000PD). All microbiological cultures positive for *Staphylococcus* or *Enterococcus* species collected during the study timeframe were evaluated. Organisms were further grouped as hospital-acquired if they were isolated from cultures collected >48 hours following admission. The all-isolate group included both hospital-acquired organisms and organisms from cultures collected <48 hours following admission. Only isolates with MIC results were included in the analysis. No changes to local microbiology laboratory testing or reporting practices of *Staphylococcus* or *Enterococcus* isolates occurred during the study time frame. The BD Phoenix™ M50 Automated Identification and Susceptibility system and PMIC-106 panel were used for susceptibility testing during the entirety of the study period. Isolates nonsusceptible to daptomycin were confirmed by E-test. The percentage of resistant isolates was calculated using 2024 Clinical & Laboratory Standards Institute (CLSI) breakpoints, except for *Enterococcus*. Since *Enterococcus* data were collected by genera, not species, the 2024 CLSI daptomycin susceptibility breakpoint for *Enterococcus faecium* of 4 µg/mL was applied to all *Enterococcus* isolates to estimate the percentage of resistant isolates.

### Statistical Analysis

Interrupted time series (ITS) analyses were utilized to approximate the effect of increased antibiotic utilization on the geometric mean (GM) MIC of each isolate grouping. The ITS design allows for fitting pre- and postinterruption time periods separately, and the estimation of the slope of the line describing the correlation between antibiotic DOT/1000PD and GM MIC during the preperiod, during the postperiod, as well as the change in slope between these respective periods. Serial correlation was assessed using autocorrelation functions, partial autocorrelation functions, and the Durbin–Watson statistic. Nonstationarity was assessed by autoregressive error modeling and seasonality was accounted for by entering a term for quarter into the models, when needed. The interruption point selected for each analysis was the month after the final policy change that impacted daptomycin or linezolid usage was implemented. For daptomycin, this was September 2021, and for linezolid, this was April 2020. Analysis was performed individually for *S. aureus* (which included both methicillin-susceptible *S. aureus* and MRSA), MRSA only, coagulase-negative staphylococci (CoNS), *Enterococcus* spp. (which included both vancomycin-susceptible *Enterococcus* spp. and VRE), and VRE only. Analysis was performed on both the all-isolate group and hospital-acquired isolate group for each organism grouping. Analyses were generated using SAS© software (SAS Institute Inc., Cary, NC, USA). All statistical comparisons were 2-tailed and a *P* value of <.05 was considered to be statistically significant.

## RESULTS

A total of 19 160 individual isolates were evaluated. Due to missing MIC data, 2571 isolates were excluded from the daptomycin analysis and 206 isolates from the linezolid analysis. *S. aureus* accounted for the majority of isolates (48.9%), followed by *Enterococcus* spp. (34.2%) and CoNS (16.9%). Methicillin resistance was present in 29.4% of *S. aureus,* and vancomycin resistance was present in 13.3% of *Enterococcus* species. Hospital-acquired organisms accounted for 37.8% of all isolates.

**Figure 1.**
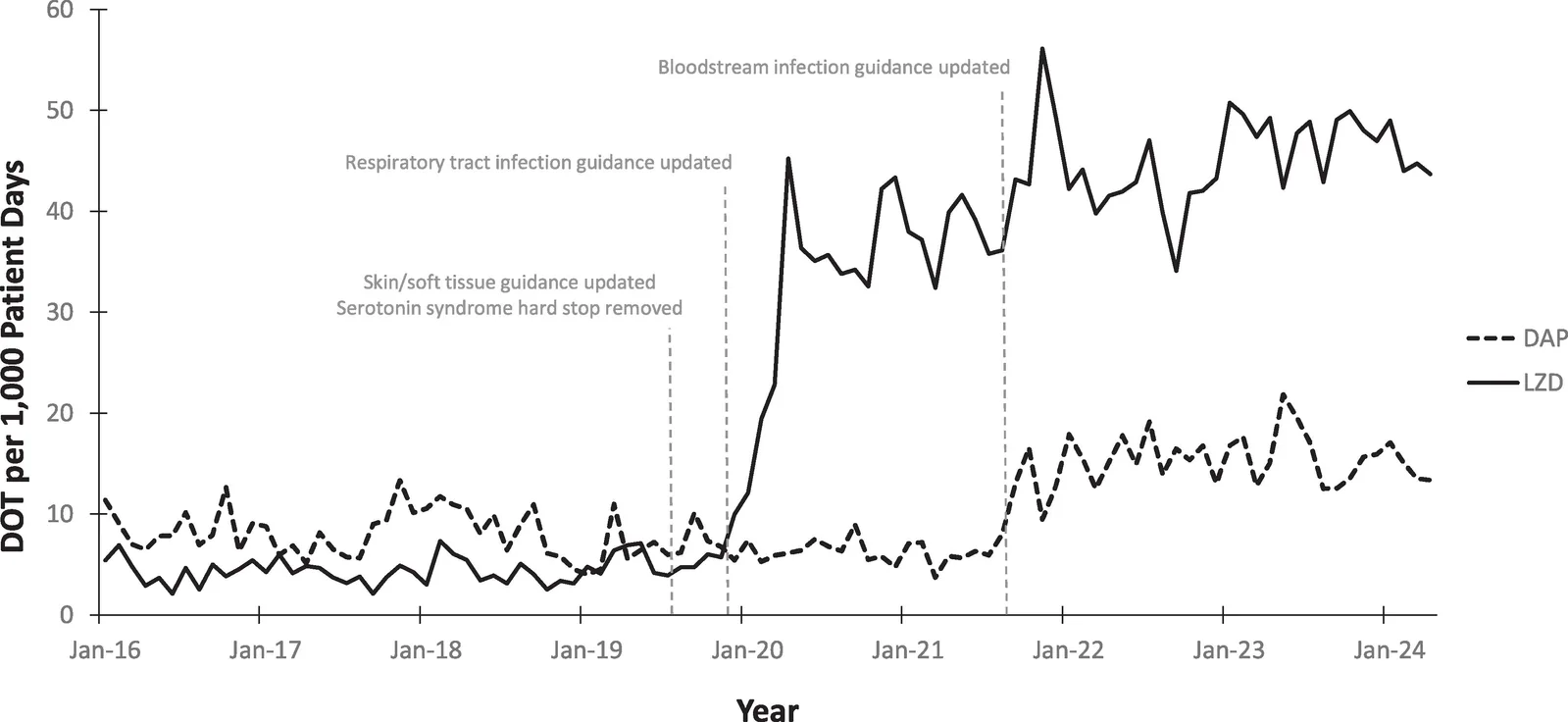
Monthly daptomycin and linezolid usage between January 2016 and April 2024. Abbreviations: DAP, daptomycin; LZD, linezolid; DOT, days of therapy.

During the study time frame, linezolid utilization increased from 4.6 DOT/1000PD prior to 2020 to 43.7 DOT/1000PD post-liberalization. Mean daptomycin utilization doubled after liberalization in 2021 from 7.6 DOT/1000PD to 15.7 DOT/1000PD (Figure 1). Additionally, vancomycin utilization declined steadily from 97.8 DOT/1000PD in 2016 to 44.4 DOT/1000PD in 2023.

### Staphylococcus aureus

**Figure 2.**
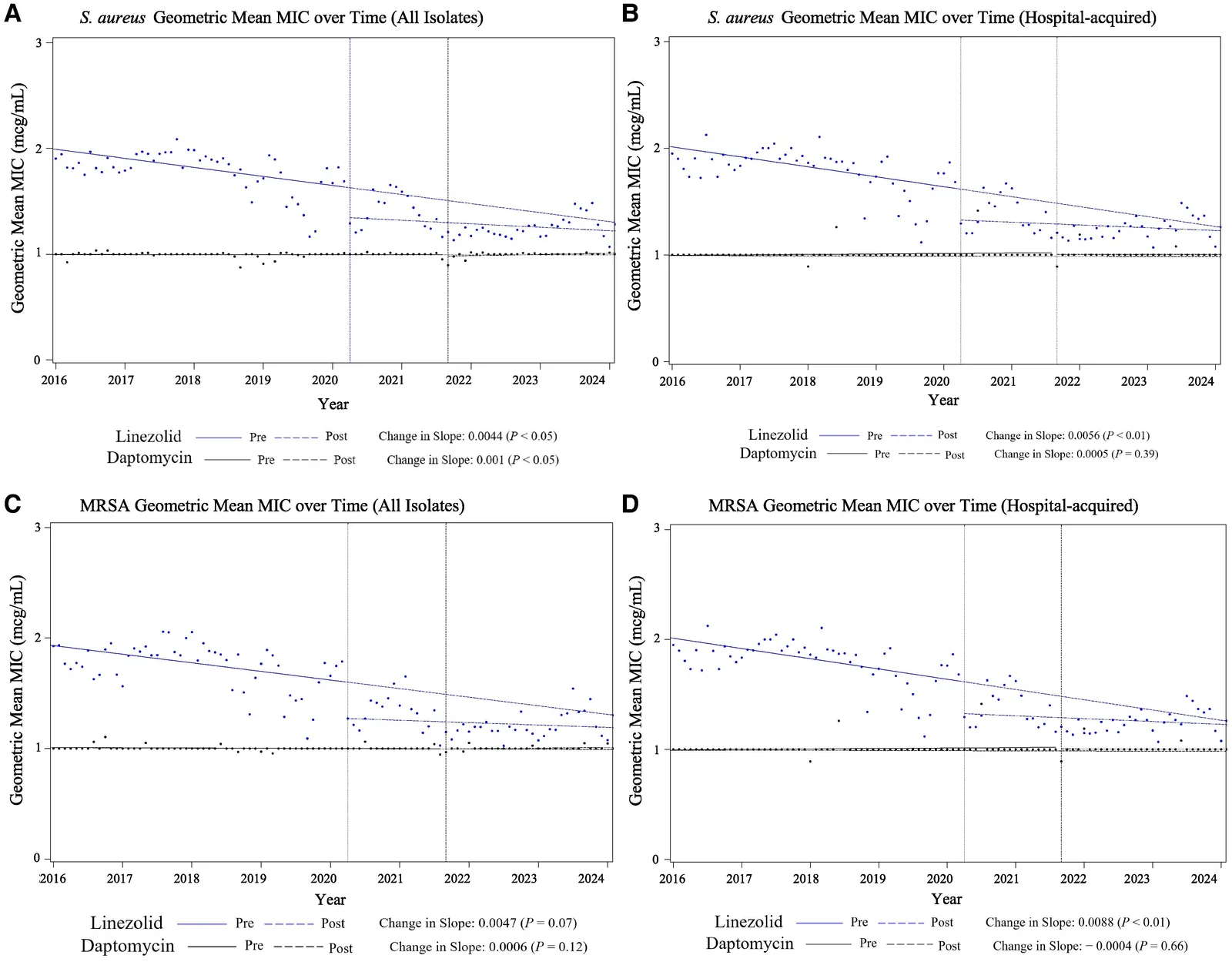
*A–D*, *Staphylococcus aureus* geometric mean minimum inhibitory concentration (MIC) over time.

No changes in daptomycin resistance rates or MIC distribution were observed in the all-isolate or hospital-acquired *S. aureus* groups, and the GM MIC and MIC_90_ remained at 1 during the entirety of the study. Over 99% of *S. aureus* isolates were susceptible to daptomycin each year based on 2024 CLSI breakpoints (Table 1). Interrupted time series analyses of the all-isolate group showed a statistically significant inversion in the daptomycin GM MIC slope after liberalization (*P* = .04) with GM MICs increasing until the end of the study period (Figure 2*A*). There was minimal change in the daptomycin GM MIC slope post-liberalization in hospital-acquired *S. aureus* isolates (*P* = .39) (Figure 2*B*).

**Table 1.** *Staphylococcus aureus* Susceptibility Characteristics by Year

	2016	2017	2018	2019	2020	2021	2022	2023	2024^a^
Daptomycin (all-isolates)									
Number of isolates	723	845	848	942	839	866	985	996	351
GM MIC (µg/mL)	1.002	1.001	0.986	0.986	1.003	0.979	1.004	1.001	1.004
MIC range (µg/mL)	0.25–4	0.5–2	0.25–2	0.125–4	1–4	0.125–2	1–4	1–4	1–4
MIC_50_ (µg/mL)	1	1	1	1	1	1	1	1	1
MIC_90_ (µg/mL)	1	1	1	1	1	1	1	1	1
Percent susceptible	99.3%	99.8%	99.9%	99.7%	99.6%	99.8%	99.6%	99.9%	99.7%
Daptomycin (hospital-acquired)									
Number of isolates	210	258	248	280	237	296	323	284	101
GM MIC (µg/mL)	0.993	1.000	0.994	0.988	1.009	0.979	1.009	1.005	1.000
MIC range (µg/mL)	0.25–1	1–1	0.5–2	0.25–4	1–4	0.25–1	1–4	1–2	1–1
MIC_50_ (µg/mL)	1	1	1	1	1	1	1	1	1
MIC_90_ (µg/mL)	1	1	1	1	1	1	1	1	1
Percent susceptible	100.0%	100.0%	99.6%	99.6%	99.2%	100.0%	99.4%	99.3%	100.0%
Linezolid (all-isolates)									
Number of isolates	929	1054	992	1094	1100	1163	1231	1267	451
GM MIC (µg/mL)	1.848	1.920	1.806	1.561	1.509	1.304	1.212	1.251	1.193
MIC range (µg/mL)	1–4	1–4	1–4	0.5–4	0.5–4	0.5–2	0.5–4	0.5–2	1–2
MIC_50_ (µg/mL)	2	2	2	2	2	1	1	1	1
MIC_90_ (µg/mL)	2	2	2	2	2	2	2	2	2
Percent susceptible	100.0%	100.0%	100.0%	100.0%	100.0%	100.0%	100.0%	100.0%	100.0%
Linezolid (hospital-acquired)									
Number of isolates	322	393	335	364	394	504	450	418	147
GM MIC (µg/mL)	1.859	1.938	1.811	1.520	1.501	1.284	1.220	1.221	1.219
MIC range (µg/mL)	1–4	1–4	1–4	1–2	0.5–2	0.5–2	1–2	0.5–2	1–2
MIC_50_ (µg/mL)	2	2	2	2	2	1	1	1	1
MIC_90_ (µg/mL)	2	2	2	2	2	2	2	2	2
Percent susceptible	100.0%	100.0%	100.0%	100.0%	100.0%	100.0%	100.0%	100.0%	100.0%

Abbreviations: MIC, minimum inhibitory concentration; GM MIC, geometric mean MIC.

^a^Data collected through April of 2024.

No *S. aureus* isolates were resistant to linezolid in this study. In the all-isolate group, the linezolid GM MIC decreased from 1.848 in 2016 to 1.251 in 2023. This declining MIC trend was also observed in hospital-acquired *S. aureus* isolates (Table 1). Interrupted time series analyses demonstrated statistically significant changes in linezolid GM MIC slope post-liberalization for the all-isolate (*P* = .03) and hospital-acquired *S. aureus* groups (*P* < .01). However, for both groups, linezolid GM MICs continued to decline after liberalization until the end of the study period (Figure 2*A* and 2*B*).

### Methicillin-Resistant *S. aureus*

No changes in daptomycin resistance rates or MIC distributions were observed in the all-isolate or hospital-acquired MRSA groups. The GM MIC and MIC_90_ remained at 1 during the entirety of the study. Over 99% of all-isolates and 97% of hospital-acquired isolates were susceptible to daptomycin each year (Table 2). Interrupted time series analysis of the data did not demonstrate significant changes in the daptomycin GM MIC slope after the liberalization in either the all-isolate (*P* = .12) or hospital-acquired MRSA groups (*P* = .66) (Figure 2*C* and 2*D*).

**Table 2.** MRSA Susceptibility Characteristics by Year

	2016	2017	2018	2019	2020	2021	2022	2023	2024^a^
Daptomycin (all-isolates)									
Number of isolates	217	265	255	276	267	240	299	301	110
GM MIC (µg/mL)	1.016	1.003	1.000	0.995	1.005	0.991	1.007	1.000	1.013
MIC range (µg/mL)	1–4	1–2	0.5–2	0.5–1	1–4	0.5–2	1–4	1–1	1–4
MIC_50_ (µg/mL)	1	1	1	1	1	1	1	1	1
MIC_90_ (µg/mL)	1	1	1	1	1	1	1	1	1
Percent susceptible	98.6%	99.6%	99.6%	100.0%	99.6%	99.6%	99.3%	100.0%	99.1%
Daptomycin (hospital-acquired)									
Number of isolates	61	88	80	86	86	94	114	83	33
GM MIC (µg/mL)	1.000	1.000	1.000	1.000	1.016	0.993	1.012	1.016	1.000
MIC range (µg/mL)	1–1	1–1	0.5–2	1–1	1–4	0.5–1	1–4	1–2	1–1
MIC_50_ (µg/mL)	1	1	1	1	1	1	1	1	1
MIC_90_ (µg/mL)	1	1	1	1	1	1	1	1	1
Percent susceptible	100.0%	100.0%	98.8%	100.0%	98.8%	100.0%	99.1%	97.7%	100.0%
Linezolid (all-isolates)									
Number of isolates	278	321	303	318	343	315	348	363	134
GM MIC (µg/mL)	1.797	1.879	1.755	1.526	1.453	1.241	1.173	1.193	1.186
MIC range (µg/mL)	1–4	1–4	1–4	0.5–4	0.5–4	0.5–2	1–4	0.5–2	1
MIC_50_ (µg/mL)	2	2	2	2	2	1	1	1	1
MIC_90_ (µg/mL)	2	2	2	2	2	2	2	2	2
Percent susceptible	100.0%	100.0%	100.0%	100.0%	100.0%	100.0%	100.0%	100.0%	100.0%
Linezolid (hospital-acquired)									
Number of isolates	100	126	107	108	132	148	141	119	48
GM MIC (µg/mL)	1.815	1.924	1.701	1.451	1.452	1.195	1.182	1.173	1.207
MIC range (µg/mL)	1–4	1–4	1–4	1–2	0.5–2	0.5–2	1–2	1–2	1–2
MIC_50_ (µg/mL)	2	2	2	2	2	1	1	1	1
MIC_90_ (µg/mL)	2	2	2	2	2	2	2	2	2
Percent susceptible	100.0%	100.0%	100.0%	100.0%	100.0%	100.0%	100.0%	100.0%	100.0%

Abbreviations: MIC, minimum inhibitory concentration; GM MIC, geometric mean MIC.

^a^Data collected through April of 2024.

No MRSA isolates were resistant to linezolid during this study. In the all-isolate group, linezolid GM MIC decreased from 1.797 to 1.193 from 2016 to 2023. Linezolid GM MICs also decreased over time in hospital-acquired isolates (Table 2). ITS analysis did not show significant changes in the linezolid GM MIC slope post-liberalization in the all-isolate group (*P* = .07). In the hospital-acquired MRSA group, there was a statistically significant change in linezolid GM MIC slope post-liberalization (*P* < .01). Although the change in trend was significant, GM MICs continued to decline after liberalization to the end of the study period (Figure 2*D*).

### Coagulase-Negative *Staphylococcus*

**Figure 3.**
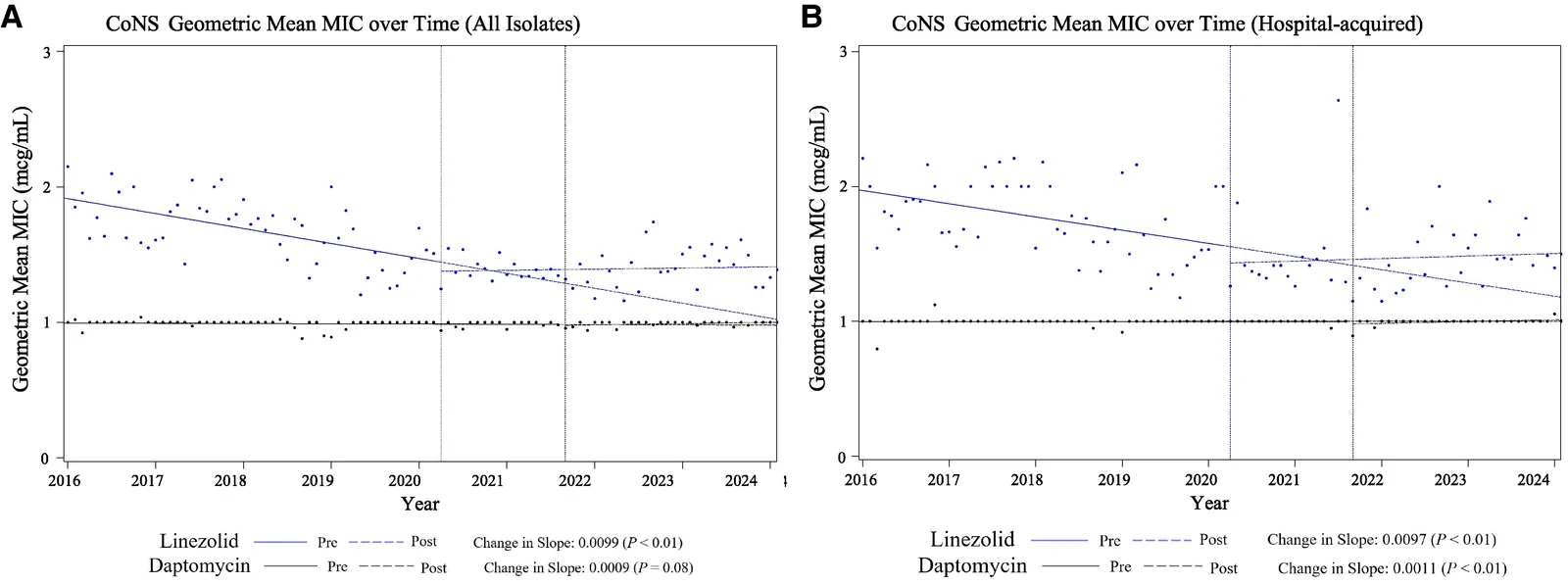
*A* and *B*, Coagulase-negative *Staphylococcus* geometric mean minimum inhibitory concentration (MIC) over time.

The daptomycin GM MIC for CoNS remained at or below the 2024 CLSI breakpoint for both the all-isolate and hospital-acquired isolate groups throughout the study. Post-liberalization, there were no CoNS isolates resistant to daptomycin (Table 3). ITS analyses of the all-isolate group did not show a significant change in the daptomycin GM MIC slope post-liberalization (*P* = .08) (Figure 3*A*). In hospital-acquired CoNS, the slope inverted post-liberalization (*P* < .01), with daptomycin GM MICs increasing to the end of the study period (Figure 3*B*).

**Table 3.** Coagulase-Negative *Staphylococcus* Susceptibility Characteristics by Year

	2016	2017	2018	2019	2020	2021	2022	2023	2024^a^
Daptomycin (all-isolates)									
Number of isolates	314	301	326	392	330	437	380	408	151
GM MIC (µg/mL)	0.998	0.998	0.979	0.988	0.987	0.981	0.995	0.997	1.000
MIC range (µg/mL)	0.25–2	0.5–1	0.125–2	0.25–2	0.25–1	0.125–1	0.25–1	0.5–1	1–1
MIC_50_ (µg/mL)	1	1	1	1	1	1	1	1	1
MIC_90_ (µg/mL)	1	1	1	1	1	1	1	1	1
Percent susceptible	99.0%	100.0%	99.7%	99.7%	100.0%	100.0%	100.0%	100.0%	100.0%
Daptomycin (hospital-acquired)									
Number of isolates	97	96	112	147	122	149	135	115	58
GM MIC (µg/mL)	0.993	1.000	0.994	0.991	1.000	0.986	1.000	1.000	1.024
MIC range (µg/mL)	0.25–2	1–1	0.5–1	0.5–2	1–1	0.5–1	1–1	1–1	1–4
MIC_50_ (µg/mL)	1	1	1	1	1	1	1	1	1
MIC_90_ (µg/mL)	1	1	1	1	1	1	1	1	1
Percent susceptible	97.9%	100.0%	100.0%	99.3%	100.0%	100.0%	100.0%	100.0%	98.3%
Linezolid (all-isolates)									
Number of isolates	356	341	341	427	332	436	376	408	154
GM MIC (µg/mL)	1.814	1.788	1.639	1.464	1.444	1.350	1.399	1.468	1.334
MIC range (µg/mL)	0.5–8	0.5–4	0.5–4	0.5–4	0.5–4	0.5–4	0.5–4	0.5–4	0.5–4
MIC_50_ (µg/mL)	2	2	2	1	2	1	1	1	1
MIC_90_ (µg/mL)	4	4	2	2	2	2	2	2	2
Percent susceptible	99.7%	100.0%	100.0%	100.0%	100.0%	100.0%	100.0%	100.0%	100.0%
Linezolid (hospital-acquired)									
Number of isolates	124	125	124	163	137	154	133	120	59
GM MIC (µg/mL)	1.860	1.882	1.645	1.511	1.491	1.459	1.440	1.542	1.460
MIC range (µg/mL)	1–4	1–4	1–4	0.5–4	0.5–4	0.5–4	0.5–4	0.5–4	0.5–4
MIC_50_ (µg/mL)	2	2	2	2	2	1	1	2	2
MIC_90_ (µg/mL)	4	4	2	2	2	2	2	2	2
Percent susceptible	100.0%	100.0%	100.0%	100.0%	100.0%	100.0%	100.0%	100.0%	100.0%

Abbreviations: MIC, minimum inhibitory concentration; GM MIC, geometric mean MIC.

^a^Data collected through April of 2024.

Only one CoNS isolate was resistant to linezolid during this study. In the all-isolate group, the linezolid GM MIC decreased from 1.814 in 2016 to 1.468 in 2023. A similar trend was observed in hospital-acquired isolates (Table 3). ITS analyses showed statistically significant inversions of the linezolid GM MIC slope for both all-isolate (*P* < .01) and hospital-acquired CoNS groups (*P* < .01). Post-liberalization, linezolid GM MICs increased until the end of the study period in both groups (Figure 3*A* and 3*B*).

### Enterococcus

**Figure 4.**
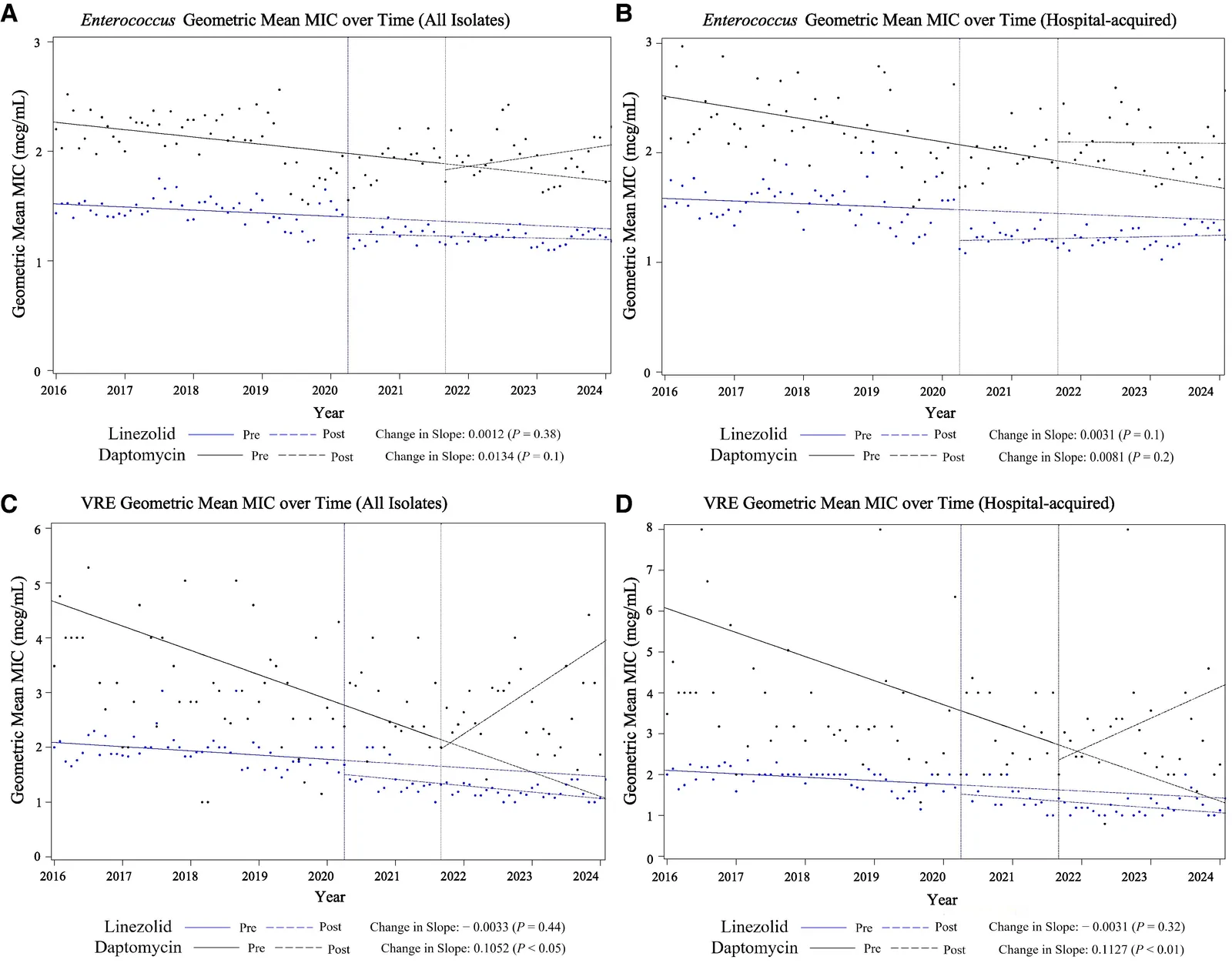
*A–D*, *Enterococcus* geometric mean minimum inhibitory concentration (MIC) over time.

The daptomycin GM MIC for the *Enterococcus* all-isolate group decreased from 2.194 to 1.742 across the observation window. This trend was also evident in hospital-acquired *Enterococcus* isolates. Resistance to daptomycin was <2% for the all-isolate group and < 4% for the hospital-acquired isolate group, based on a breakpoint of 4 µg/mL (Table 4). Interrupted time series analyses showed no significant changes in the daptomycin GM MIC slope in either the all-isolate (*P* = .99) or hospital-acquired *Enterococcus* groups (*P* = .19) after liberalization (Figure 4*A* and 4*B*).

**Table 4.** *Enterococcus* Susceptibility Characteristics by Year

	2016	2017	2018	2019	2020	2021	2022	2023	2024^a^
Daptomycin (all-isolates)									
Number of isolates	636	640	753	787	791	750	759	782	257
GM MIC (µg/mL)	2.194	2.214	2.221	1.884	1.823	1.993	2.022	1.742	2.060
MIC range (µg/mL)	1–32	0.5–512	0.125–512	0.25–64	0.25–256	0.5–16	0.125–16	0.125–32	0.06–512
MIC_50_ (µg/mL)	2	2	2	2	2	2	2	2	2
MIC_90_ (µg/mL)	4	4	4	4	4	4	4	4	4
Percent susceptible	98.3%	98.6%	98.9%	98.2%	99.1%	99.3%	99.1%	99.3%	97.3%
Daptomycin (hospital-acquired)									
Number of Isolates	257	240	307	283	327	327	303	332	106
GM MIC (µg/mL)	2.396	2.387	2.306	2.060	1.962	2.092	2.163	1.931	2.180
MIC range (µg/mL)	1–32	0.5–512	1–512	0.25–64	0.25–256	0.5–16	0.25–16	1–32	0.06–512
MIC_50_ (µg/mL)	2	2	2	2	2	2	2	2	2
MIC_90_ (µg/mL)	4	4	4	4	4	4	4	4	4
Percent susceptible	96.1%	97.1%	98.7%	97.5%	98.8%	99.1%	99.3%	99.4%	99.0%
Linezolid (all-isolates)									
Number of isolates	790	788	818	782	790	754	763	772	245
GM MIC (µg/mL)	1.463	1.529	1.489	1.355	1.284	1.228	1.222	1.129	1.192
MIC range (µg/mL)	0.5–8	0.5–128	0.5–256	0.5–4	0.5–128	0.5–2	0.5–2	0.5–4	0.5–2
MIC_50_ (µg/mL)	2	2	2	1	1	1	1	1	1
MIC_90_ (µg/mL)	2	2	2	2	2	2	2	2	2
Percent susceptible	96.5%	96.4%	97.8%	99.9%	99.9%	100.0%	100.0%	99.7%	100.0%
Linezolid (hospital-acquired)									
Number of isolates	338	318	334	288	331	334	303	329	99
GM MIC (µg/mL)	1.538	1.598	1.542	1.384	1.307	1.220	1.226	1.137	1.225
MIC range (µg/mL)	0.5–8	1–32	0.5–8	0.5–4	0.5–64	0.5–2	0.5–2	0.5–2	0.5–2
MIC_50_ (µg/mL)	2	2	2	1	1	1	1	1	1
MIC_90_ (µg/mL)	2	2	2	2	2	2	2	2	2
Percent susceptible	95.0%	96.2%	97.1%	99.7%	99.7%	100.0%	100.0%	100.0%	100.0%

Abbreviations: MIC, minimum inhibitory concentration; GM MIC, geometric mean MIC.

^a^Data collected through April of 2024.

The linezolid GM MIC for the *Enterococcus* all*-*isolates group decreased from 1.463 to 1.129 during the study period. This trend was again evident in hospital-acquired isolates. Over 95% of *Enterococcus* isolates were susceptible to linezolid (Table 4). Interrupted time series analyses showed no significant changes in the linezolid GM MIC slope in either the all-isolate (*P* = .38) or hospital-acquired *Enterococcus* groups (*P* = .1) post-liberalization (Figure 4*A* and 4*B*).

### Vancomycin-Resistant *Enterococcus*

The daptomycin GM MIC for the VRE all-isolates group decreased from 4.112 in 2016 to 2.197 in 2023. This trend was also apparent in hospital-acquired VRE. Daptomycin susceptibility increased over time from 82% to 98.3% in all-isolate and 78.4% to 97.3% in hospital-acquired isolates, based on a breakpoint of 4 µg/mL (Table 5). Interrupted time series analyses showed statistically significant daptomycin GM MIC slope inversions for both all-isolate (*P* = .01) and hospital-acquired VRE groups (*P* < .01) post-liberalization with GM MICs increasing until the end of the analysis for both groups (Figure 4*C* and 4*D*).

**Table 5.** Vancomycin-resistant *Enterococcus* Susceptibility Characteristics by Year

	2016	2017	2018	2019	2020	2021	2022	2023	2024^a^
Daptomycin (all-isolates)									
Number of isolates	50	44	100	97	84	86	100	92	24
GM MIC (µg/mL)	4.112	4.000	3.160	2.514	2.852	2.486	2.657	2.197	2.594
MIC range (µg/mL)	1–32	1–512	1–512	0.25–32	1–256	1–16	0.25–16	1–32	1–512
MIC_50_ (µg/mL)	4	4	4	2	2	2	2	2	2
MIC_90_ (µg/mL)	8	8	4	4	4	4	4	4	4
Percent susceptible	82.0%	84.1%	94.0%	91.8%	95.2%	97.7%	95.0%	98.3%	95.8%
Daptomycin (hospital-acquired)									
Number of isolates	37	34	59	53	50	46	59	58	12
GM MIC (µg/mL)	4.735	4.709	3.315	3.120	3.249	2.828	2.845	2.367	4.000
MIC range (µg/mL)	1–32	1–512	1–512	1–32	1–256	1–16	0.25–16	1–32	1–512
MIC_50_ (µg/mL)	4	4	4	4	4	4	4	2	2
MIC_90_ (µg/mL)	16	32	4	8	4	4	4	4	4
Percent susceptible	78.4%	79.4%	94.9%	88.7%	92.0%	95.7%	93.2%	97.3%	91.7%
Linezolid (all-isolates)									
Number of isolates	149	130	148	86	71	85	93	85	23
GM MIC (µg/mL)	1.954	2.098	1.991	1.662	1.598	1.246	1.169	1.182	1.062
MIC range (µg/mL)	0.5–4	1–128	1–256	0.5–2	1–2	1–2	1–2	1–2	0.5–2
MIC_50_ (µg/mL)	2	2	2	2	2	1	1	1	1
MIC_90_ (µg/mL)	4	2	2	2	2	2	2	2	2
Percent susceptible	89.9%	90.8%	96.6%	100.0%	100.0%	100.0%	100.0%	100.0%	100.0%
Linezolid (hospital-acquired)									
Number of isolates	94	76	88	51	39	46	54	51	11
GM MIC (µg/mL)	2.030	2.018	1.938	1.653	1.645	1.216	1.094	1.215	1.134
MIC range (µg/mL)	1–4	1–16	1–8	1–2	1–2	1–2	1–2	1–2	1–2
MIC_50_ (µg/mL)	2	2	2	2	2	1	1	1	1
MIC_90_ (µg/mL)	4	2	2	2	2	2	2	2	2
Percent susceptible	89.4%	93.4%	96.6%	100.0%	100.0%	100.0%	100.0%	100.0%	100.0%

Abbreviations: MIC, minimum inhibitory concentration; GM MIC, geometric mean MIC.

^a^Data collected through April of 2024.

The linezolid GM MIC for the VRE all-isolates group decreased from 1.954 to 1.182 across the 8-year period. This trend was also observed in the hospital-acquired VRE group. Linezolid susceptibility rates increased over time, and no VRE isolates were resistant to linezolid after liberalization (Table 5). Interrupted time series analyses showed no significant changes in the linezolid GM MIC slope in either the all-isolate (*P* = .44) or hospital-acquired VRE groups (*P* = .32) post-liberalization (Figure 4*C* and 4*D*).

## DISCUSSION

Our study did not observe any concerning trends in *Staphylococcus* or *Enterococcus* resistance rates or MIC distributions over an 8-year period that included a 3-year follow-up after liberalization of daptomycin and linezolid use. Clinically relevant resistance was not prevalent in this study, and increased daptomycin and linezolid utilization was not associated with increasing MICs among MRSA, *Enterococcus* spp., and hospital-acquired *S. aureus*. Furthermore, the declining linezolid MIC trend observed in *S. aureus* and *Enterococcus* spp. persisted even after liberalization. In contrast, subclinical increases in daptomycin MICs were observed among VRE and hospital-acquired CoNS post-liberalization. Subclinical increases in MICs were also observed in CoNS after liberalization of linezolid. Although the increasing MIC trends observed among hospital-acquired CoNS isolates were statistically significant for both drugs, the magnitude of change over time was minimal, and MICs remained well below clinically relevant resistance thresholds throughout the study period. Additionally, while extrapolation of MIC trajectories is limited by the relatively short post-liberalization observation period, the minimal upward MIC trends observed in CoNS suggest longitudinal stability in susceptibility to both daptomycin and linezolid. Among hospital-acquired VRE, however, the increasing daptomycin MIC trend was more pronounced and may indicate that daptomycin susceptibility in VRE is less durable over time than in CoNS. While the increasing MICs in hospital-acquired VRE were a statistically significant finding, it should be noted that this observation is based on a small number of isolates collected over 8 years. Notably, we did not demonstrate any significant increase in MICs post-liberalization for hospital-acquired *S. aureus* isolates.

Resistance to daptomycin and linezolid was rare in this study. Less than 1% of all-isolates were resistant to either agent according to 2024 CLSI breakpoints, which is consistent with prior reports. A meta-analysis of studies spanning a 30-year time frame reported daptomycin and linezolid resistance rates of 0.1%–0.3% in *Staphylococcus* isolates [37]. The European Antimicrobial Resistance Surveillance Network also reported static rates of antibiotic resistance in *S. aureus* and *E. faecium* [36]. Other studies have shown similar results, including resistance rates as high as 2.9% for *Enterococcus* species [42]. In the present study, our daptomycin and linezolid resistance rates in *Enterococcus* spp. were 1.25% and 1.18%, respectively. Since we did not collect species-level *Enterococcus* data, the daptomycin resistance rate is likely an overestimation because the more conservative *E. faecium* breakpoint of 4 µg/mL was applied to any non-*E. faecium* species collected during the study period.

Although epidemiologic reports do not suggest clinically concerning trends in Gram-positive resistance, some studies have demonstrated relationships between antibiotic consumption and subsequent development of resistance. One retrospective analysis of over 2000 CoNS isolates reported a nearly 3-fold increase in daptomycin consumption over a 12-month period that was correlated with an increase in daptomycin resistance from 0.9% to 6% [38]. Another small retrospective study found a similar relationship between increased linezolid usage and subsequent resistance in CoNS. The authors reported a 4.9% decrease in susceptibility across 2 years that followed a 51% increase in linezolid consumption during the same time span [41]. Other studies and case reports corroborate the relationship between antibiotic exposure and resistance in *Staphylococcus* and *Enterococcus* species [39–41, 43–47]. While our study did demonstrate a statistically significant association between antibiotic utilization and increasing MICs in CoNS and VRE, we observed no clinically meaningful changes in resistance rates or MIC distributions across 8 years of data. Differences in antibiotic prescribing practices and the length of observation could explain the variance in results from prior literature.

An unexpected observation from our data was the significant decline in linezolid MICs that persisted in *S. aureus* isolates through the end of the study period. Cross-resistance between clindamycin and linezolid is a possible explanation for this decreasing linezolid MIC trend. Mutations in 23S rRNA of *S. aureus* and *Enterococcus* spp. appear to be the most prevalent cause of linezolid resistance [48]. The concentration of 23S rRNA gene copies carrying a G2576T mutation is closely correlated to the degree of linezolid resistance [49, 50]. The G2576T mutation in the 23S rRNA genes of *S. aureus* also confers high-level cross-resistance to clindamycin as well as erythromycin and gentamicin [51]. During the 8 years of this study, clindamycin use at UCH decreased from 12 to 2 DOT/1000PD. We theorize that the decrease in clindamycin utilization reduced selective pressure in *S. aureus* and therefore the frequency of 23S rRNA gene mutations within the population. Further research including genomic sequencing is required to investigate this theory and was thus beyond the scope of the current study. Additionally, this hypothesis does not explain the overall decline in daptomycin MICs that was observed in *Enterococcus*.

One strength of the current study is that it differentiates hospital-acquired isolates from community-acquired isolates. This addresses the concern that data from community-acquired organisms do not reflect the full impact of antibiotic exposure on a population within a health system. For example, a study by Markwart et al [36] reported higher rates of daptomycin and linezolid resistance in isolates that were collected from an intensive care unit compared to other settings. Similarly, in the present study, hospital-acquired isolates were more resistant than community-acquired isolates at baseline. However, rising daptomycin and linezolid MICs were not observed exclusively in hospital-acquired isolates. Statistically significant MICs increases were also seen in the all-isolate groups of *S. aureus*, CoNS, and VRE. Since all-isolate groups also contained hospital-acquired isolates, it is difficult to quantify the true impact of daptomycin and linezolid liberalization on resistance in the community. Furthermore, the evaluation of treatment-emergent resistance within specific patients was not assessed in this study. Despite these limitations, since susceptibility rates did not decrease throughout the collection period, we suspect any effect on resistance in the community has been negligible. Another strength of this study is the prevalent use of both daptomycin and linezolid at UCH. Several previous studies utilized defined daily doses (DDD) to describe antibiotic consumption. We utilized DOT/1000PD due to data accessibility and decreased intra-patient variability in usage reporting compared to DDD. Although direct comparison between DDD and DOT/1000PD can be problematic [52], there is substantial variation in the extent of daptomycin and linezolid consumption across studies that reported prescribing data, and the utilization of these agents at UCH appears to be relatively high [38, 42, 43]. In contrast, Li et al [40] reported linezolid utilization of around 100 DOT/1000PD toward the end of their study period, which is more than double the observed linezolid usage at our site. It is thus possible that greater daptomycin and linezolid utilization is necessary to select for observable increases in MICs.

This study and the data generated from it have limitations. First, this study is limited by its retrospective design, which may introduce bias related to an inability to properly account for any additional confounders that may have impacted either antibiotic utilization or pathogen susceptibilities in the data analysis. Prospective studies are warranted to validate these findings and establish causality. Second, we collected isolates and MIC data from a single hospital. Our laboratory does not routinely run susceptibility testing on CoNS isolated from nonsterile specimens or blood cultures with growth in only one set. Additionally, any organisms isolated from mixed cultures are only worked up when there are 3 or fewer distinct colonies in the sample. Although excluding these isolates may have restricted our ability to identify meaningful changes in resistance over time, we do not believe this is a significant limitation. By excluding probable contaminants, our large data set reflects a greater proportion of clinically relevant isolates. A third limitation of this study is the absence of source-stratified analyses, as specimen source data was not collected. Pooling isolates across infection types may have reduced the sensitivity of our analysis to detect MIC changes, particularly for indications where antibiotic use was most directly affected by liberalization (eg, bloodstream infections). Another potential limitation is the relatively short observational period following the increase in daptomycin and linezolid utilization at UCH. Prior studies have observed changes in resistance occurring within only 2 years of increased antibiotic consumption [38, 40, 42]. Our study analyzed isolates over an 8-year period including 31 months and 47 months of data collected following the liberalization of daptomycin and linezolid, respectively. Conversely, 1 single-center study reported a 1.5-fold increase in linezolid usage that preceded an increase in isolation of linezolid-resistant VRE by several years [41]. Although the authors did not specifically analyze the relationship between antibiotic use and incidence of linezolid-resistant VRE, the increase in linezolid resistance occurred across an 8-year period after the increase in linezolid use. Therefore, it is possible that a longer observation period after increasing utilization is necessary to identify significant changes in resistance reported in some studies.

Limitations notwithstanding, these data have several implications for practice. As previously mentioned, daptomycin and linezolid have established superiority over vancomycin for a variety of clinical scenarios. Historically, the broader spectrum of activity and higher acquisition costs of daptomycin and linezolid compared to vancomycin may have deterred their use. Our results suggest that the liberalization of daptomycin and linezolid did not result in subsequent increases in resistance rates among several relevant pathogens. However, the observed subclinical MIC trend noted among CoNS and VRE is worth confirming with continued monitoring and prospective studies. The apparent low risk of developing resistance and minimal epidemiological impact of increased daptomycin and linezolid use observed in this study could potentially prompt a re-evaluation of local prescribing practices at other health systems.
